# Primary malignant myelomatous pleural effusion

**DOI:** 10.1002/ccr3.634

**Published:** 2016-07-15

**Authors:** Ankit Mangla, Nikki Agarwal, George J. Kim, Rosalind Catchatourian

**Affiliations:** ^1^ Division of Hematology / OncologyDepartment of Internal MedicineJohn H. Stroger Jr. Hospital of Cook County1901 West Harrison StreetChicagoIllinois60612USA; ^2^ Division of Hematology / OncologyJohn H. Stroger Jr. Hospital of Cook County1901 West Harrison StreetChicagoIllinois60612USA; ^3^ Division of Surgical PathologyDepartment of PathologyJohn H. Stroger Jr. Hospital of Cook CountyUniversity of Illinois at Chicago1901 West Harrison StreetChicagoIllinois60612USA

**Keywords:** Mott cells, multiple myeloma, pleural effusion, Russell bodies

## Abstract

Primary malignant myelomatous pleural effusion (PMMPE) occurs in less than 1% of patients with multiple myeloma and is diagnosed either by visualization of plasma cells on cytology or by positive flow cytometry. The presence of immature plasma cells characterized by high nucleus to cytoplasm ratio, visible nucleolus and presence of Mott cells and Russell bodies are independent poor prognostic factors. The clinician should differentiate PMMPE from secondary pleural effusion as it is associated with a significantly worse prognosis and poor overall survival.

## Introduction

Malignant pleural effusion in a patient with multiple myeloma (MM) is most commonly a consequence of end‐organ damage caused by the monoclonal gammopathy and is seen in up to 6% patients 1, 2. Primary malignant myelomatous pleural effusion (PMMPE) is pleural effusion in a patient with MM which occurs due to the myelomatous involvement of the pleura or of the surrounding structures which eventually invade into the pleural cavity 1, 2. The prognosis of patient with PMMPE is very poor 1. The morphology of the plasma cells also have a bearing on the prognosis of the patient where plasmablastic differentiation has been shown to be associated with poor prognosis 3. In this case report, we will discuss the incidence and presentation of PMMPE, morphology of immature plasma cells and mott cells with Russell bodies.

## Case Report

**Figure 1:**
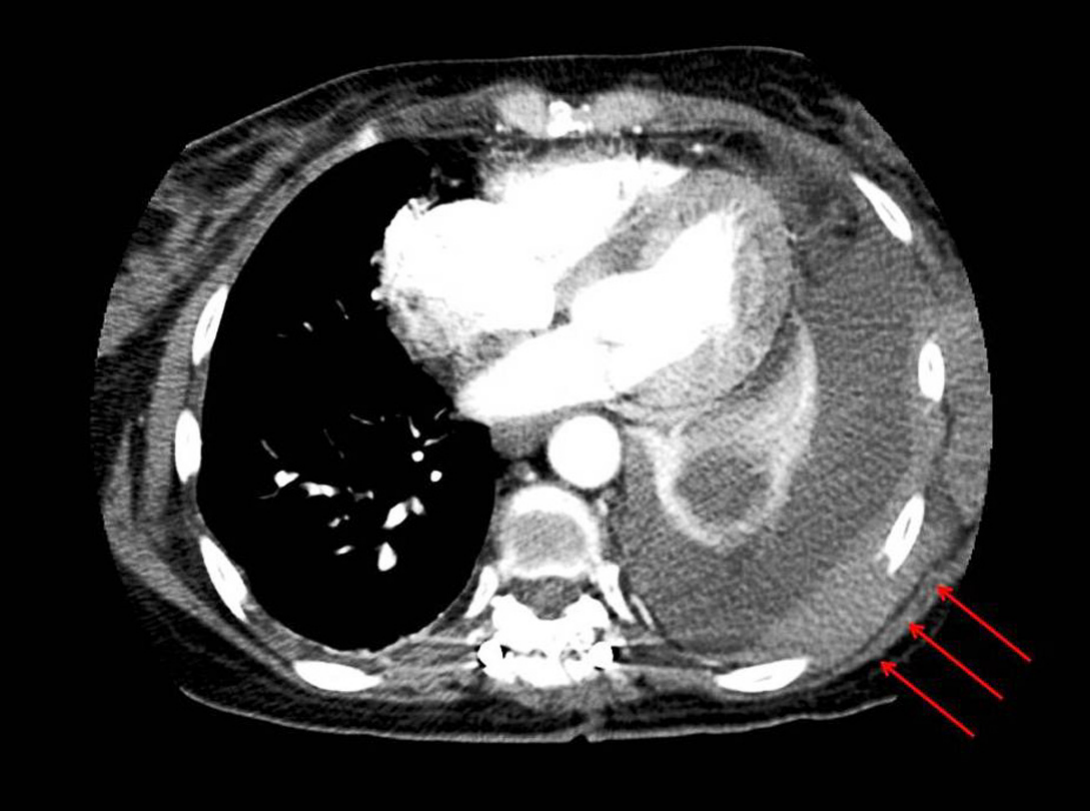
CT chest showing large pleural effusion with collapsed lobe of the lung, right mediastinal shift, and pleural thickening due to metastasis to pleura (marked by red arrows).

**Figure 2:**
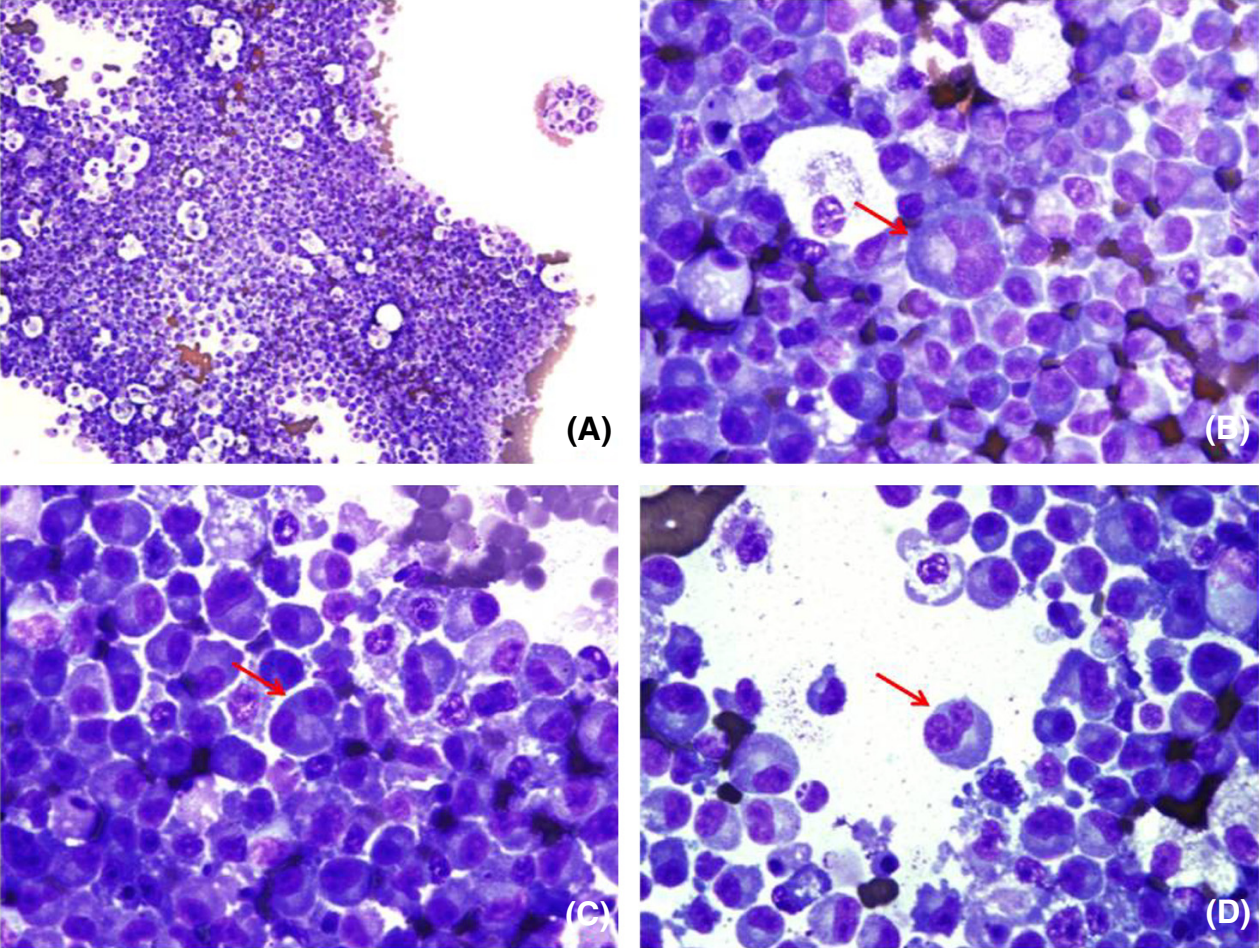
Panel A – Numerous plasma cells seen in the pleural effusion. Panel B – Multilobed plasma cell. Panel C – Bilobed plasma cell. Panel D – Numerous immature plasma cells (multilobed nucleoli, visible nucleolus, high nuclear to cytoplasmic ratio).

**Figure 3:**
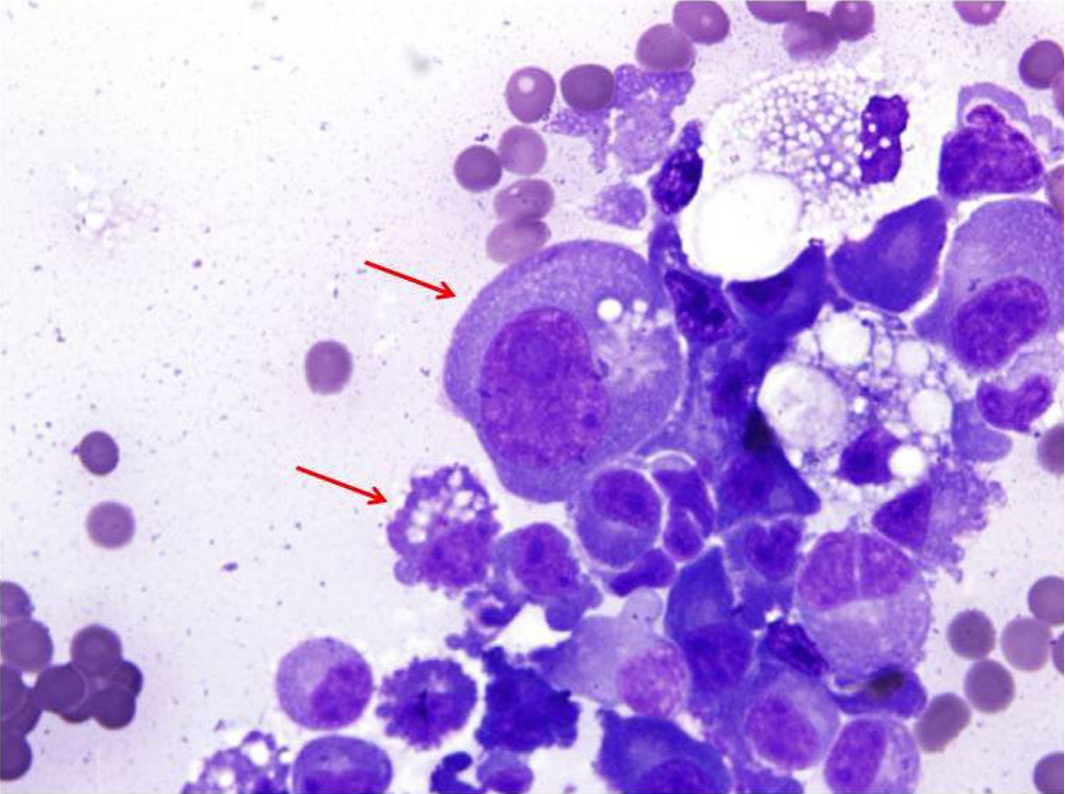
Mott cells: Russell bodies seen within the cytoplasm as vesicles and dilated endoplasmic reticulum cisternae.

A middle aged woman, recently diagnosed with IgG‐Kappa (IgGk) MM presented with increasing shortness of breath after three cycles of induction chemotherapy. She had initially presented with a compression fracture of T6 vertebrae causing spinal cord compression which was managed with emergent laminectomy and pedicle screw fixation of the spine. Magnetic resonance imaging (MRI) of thoracic spine showed an epidural mass extending from T4 to T8 vertebrae which was biopsied and histopathology of the mass showed numerous plasma cells positive for CD138 (Syndecan‐1) and Kappa light chain restriction. A bone marrow (BM) biopsy showed 30% plasma cells based on CD 138 staining, intact trilineage hematopoiesis, and normal cellularity for age. Cytogenetic analysis of the BM tissue showed a normal karyotype (46XX) and Fluorescence (FISH) studies showed t(4;14) and monosomy 13 indicating poor prognosis. Normal signal patterns were detected for p53 tumor suppressor gene and cyclin D1 and normal ploidy was observed for chromosomes 5, 9, and 15. She was started on induction chemotherapy with novel agents comprising a combination of bortezomib, cyclophosphamide, and dexamethasone, while being evaluated for autologous stem cell transplant. She had also received 3000 Gy of external beam radiation therapy (EBRT) to the epidural mass prior to starting her chemotherapy. After three cycles of induction chemotherapy, the patient started experiencing dyspnea on exertion which worsened over 1 week, limiting her exercise tolerance to a few steps. She denied any other symptoms suggestive of a cardiac etiology. Imaging of the chest showed a new large pleural effusion of the left side with left lung atelectasis, right mediastinal shift, and metastasis to the pleura (Fig. 1). The patient underwent therapeutic thoracentesis and pleural fluid (PF) was sent for sent for cytological and biochemical analysis. The biochemical analysis showed an exudative pleural effusion by Light's criteria (fluid/serum protein ratio more than 0.5, serum/fluid LDH ratio > 0.6 and fluid LDH more than 2/3rd the upper limit for normal LDH). The PF protein was 8.5 g/dL (serum protein‐10.5 g/dL), PF Albumin was 1.9 g/dL, PF Glucose was 79 mg/dL and PF lactate dehydrogenase (LDH) level was 407 U/L (Serum LDH‐166 U/L). Cytology of the fluid showed numerous plasma cells with numerous immature bilobed and multilobed plasma cells (Fig. 2) along with Mott cells (Fig. 3). The kappa and Lambda in situ hybridization studies were noncontributory. The fluid reaccumlated within 3 weeks and required a second therapeutic thoracentesis and eventually multiple thoracentesis. The patient had high extramedullary disease burden which could not be controlled with multiple chemotherapy regimens and she eventually died after nearly 8 months of therapy.

## Discussion

The incidence of pleural effusion in patients diagnosed with MM is nearly 6%; however, this is mostly due to secondary causes (like heart failure, renal failure etc.) which could be a complication of the myeloma or the monoclonal gammopathy due to underlying myeloma 1, 2. Primary malignant myelomatous pleural effusion, which is a direct result of the underlying myeloma itself, occurs in less than 1% patients diagnosed with MM 1, 2. Primary malignant myelomatous pleural effusion is called primary as it occurs due to direct extension of the plasma cells from adjacent chest wall lesions or pulmonary lesions, hematogenous spread, or due to lymphatic obstruction 1. Left‐sided PMMPE is more common than right side involvement and it tends to be associated more with IgA MM, which in turn is associated more with del(13) 1. In general, hypodiploid groups (monosomy 13, del 13, del 17, 1q gain) with t(4;14)(p16;q32) and t(14;16)(q32;q23) are considered poor prognostic groups, while hyperdiploid patients with t(11;14)(q13;q32) have better prognosis 4. A total of 50% of patients have aberrations with chromosome 13, out of which 85% have monosomy 13 and 15% have del (13). Recent evidence has shown the close association between monosomy 13 and t(4;14)(p16;q32) which foretells a poor prognosis as seen in our patient 4. However, patients who develop PMMPE have also been known to have poor prognosis, but it is not clear whether this due to the PMMPE itself or poor prognosis due to genetic mutations 1. The plasma cells tend to be immature as demonstrated by multilobed nucleus, nucleus to cytoplasmic ratio of less than 2:1 or nearly 1:1, loose reticular chromatin and multiple nucleoli (Fig. 2). Morphologic subtypes have been shown to be important prognostic factors, with a trend toward immaturity strongly associated with poor prognosis 3. Mott cells, named after the surgeon F. W. Mott, are plasma cells with spherical inclusion bodies (Fig. 3) 5. The inclusion bodies are also called Russell bodies, after William Russell, who had described them in 1890, however, was unable to explain their significance. The spherical inclusion bodies are now known to comprise of mutated immunoglobulins (which is neither secreted nor degraded) within a vesicular structure which is in turn derived from endoplasmic reticulum 5. Mott cells are characterized by expression of B220, CD5, CD43, and CD 11b and their biogenesis has been linked to a genetic locus‐microsatellite marker (D4 Mit 70 and D4 Mit 48) which is in close proximity to lmh‐1‐ another locus responsible for hypergammaglobulenemia 5. Mott cells and Russell bodies are found in various pathological conditions including reactive plasmacytosis, lymphoid malignancies, multiple myeloma, and nonmalignant conditions like Wiskott–Aldrich syndrome and Von Recklinghausen's neurofibromatosis 4. Special stains like Periodic Acid‐Schiff (PAS) and May‐Grünwald‐Giemsa (MGG) stain can be used to highlight Mott cells, however, there appearance is very characteristic under the microscope as shown in Figure 3. To conclude, primary malignant PMMPE is patients with MM is very rare and occurs in less than 1% of patients. However, it is imperative to be aware of this clinical entity as it is separate from pleural effusion which occurs in a patient with MM due to numerous other secondary causes (like heart failure or renal failure) which may be a direct consequence of underlying myeloma. Primary malignant myelomatous pleural effusion is associated with poor prognosis and should alert the clinician toward the aggressive nature of the underlying myeloma.

## Conflicts of Interest

None declared.
